# Autonomic control of cardiac rhythm at the onset of muscle metaboreflex activation following exercise in healthy young and old adults

**DOI:** 10.14814/phy2.71009

**Published:** 2026-07-06

**Authors:** Tyler E. Oliver, Miguel E. Sánchez‐Hechavarría, Ramón Carrazana‐Escalona, Cheryl A. Blaha, Lawrence I. Sinoway, Rachel C. Drew

**Affiliations:** ^1^ Department of Exercise and Health Sciences University of Massachusetts Boston Boston Massachusetts USA; ^2^ Departamento de Ciencias Básicas, Facultad de Medicina Universidad Católica de la Santísima Concepción Concepción Chile; ^3^ Facultad de Ciencias de la Salud Universidad Adventista de Chile Chillán Chile; ^4^ Penn State Heart and Vascular Institute Penn State College of Medicine Hershey Pennsylvania USA

**Keywords:** age, heart rate variability, muscle metaboreflex

## Abstract

Sympathetic and parasympathetic nervous system (SNS, PNS) control of cardiac rhythm during muscle metaboreflex activation (MMA) are important components of the physiological response to exercise but poorly understood. Therefore, SNS and PNS control of cardiac rhythm at the onset of MMA following exercise was examined in healthy young and old adults. Fourteen young (26 ± 2 years) and 10 old (62 ± 3 years) healthy adults performed 90‐s, one‐legged, isometric, calf exercise at 70% maximal voluntary contraction (MVC) and underwent a 0% MVC control trial, with unilateral circulatory occlusion beginning 5 s before exercise cessation and maintained for 30 s following exercise to isolate MMA. Absolute and normalized low‐frequency (aLF, nLF) and high‐frequency (aHF, nHF) bands and LF/HF were analyzed during a 30 s baseline period and the first 30 s of MMA following exercise (70% MVC) or continued rest (0% MVC). A time‐trial interaction in LF/HF was observed in young adults (*p* = 0.043), with LF/HF elevated during 0–10 s of MMA compared to 10–30 s during the 70% MVC trial. This interaction was not observed in old adults (*p* = 0.490). These results indicate that healthy young adults exhibit restoration of PNS control of cardiac rhythm at the onset of MMA following exercise, whereas this restoration is absent in healthy old adults.

## Graphical abstract

Healthy young adults exhibit restoration of parasympathetic nervous system control of cardiac rhythm at the onset of muscle metaboreflex activation following exercise. Conversely, this restoration of parasympathetic nervous system control of cardiac rhythm at the onset of muscle metaboreflex activation following exercise is absent in healthy old adults.

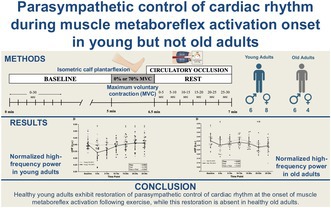

## 1 INTRODUCTION

During volitional exercise, the autonomic nervous system (ANS) provides a concerted and targeted response to meet the rapid and substantial cardiovascular and metabolic demands imposed by the working skeletal muscles. Several neural mechanisms are activated from simultaneous feed‐forward (central command) and feedback (exercise pressor) inputs to the cardiovascular control center in the medulla oblongata in the brainstem to cause rapid neural adjustments in response to the level of these demands (Fisher et al., 2015). At the immediate cessation of exercise, removal of central command and muscle mechanoreflex inputs that were present during exercise cause abrupt reflex alterations in sympathetic nervous system (SNS) and parasympathetic nervous system (PNS) activities, allowing the body to eventually return to a resting state following exercise performance (Michael et al., 2017). However, depending on the exercise performed, muscle metaboreflex activation (MMA) can occur, e.g., such as with isometric exercise, that remains even after the end of exercise performance, competing against alterations of autonomic outflow to reduce heightened SNS activity and restore PNS activity by removing inhibitory inputs (Fisher et al., 2015; Michael et al., 2017).

Heart rate variability (HRV) is a non‐invasive approach for assessing ANS activity that can provide insight into PNS and SNS contributions to the control of cardiac rhythm (Michael et al., 2017). Short‐term (e.g., 5 min) or long‐term (e.g., 24 h) electrocardiogram (ECG) recordings have been used to perform time‐domain HRV analyses involving time intervals between successive heartbeats or frequency‐domain HRV analyses involving identification of frequency components within the physiological waveforms (Shaffer & Ginsberg, 2017; Task Force Report, 1996). For these time‐ and frequency‐domain analyses, data have needed to be stationary, meaning that the physiological signal is stable with constant variance over time. Continuous wavelet transform (CWT) is an HRV analysis approach that allows time‐frequency analysis in which short‐duration windows are used to analyze high frequencies and long‐duration windows are used to analyze low frequencies (Davrath et al., 2006; Gamero et al., 2002; Toledo et al., 2003). This approach enables frequency components to be extracted instantaneously from the signal across time, so information reflecting both SNS and PNS activity can be gathered from a non‐stationary signal, a physiological signal with rapidly fluctuating characteristics. Therefore, CWT allows analysis of HRV at times of rapid autonomic adjustments that is not possible with other HRV analysis techniques (Davrath et al., 2006; Gamero et al., 2002; Oliver et al., 2023; Pichot et al., 1999; Toledo et al., 2003).

MMA occurs due to accumulation of metabolites produced by working skeletal muscle that stimulates afferent neural pathways, causing sympathetically mediated increases in both blood pressure (BP) and heart rate (HR) (Iellamo et al., 1999; O'Leary, 1993). Following isometric exercise, MMA can remain, resulting in continued SNS activation while PNS activity drastically increases to restore HR to resting levels as well as in an attempt to decrease the MMA‐induced elevated BP through the arterial baroreflex (Fisher et al., 2010). Some studies have investigated these competing parallel mechanisms following the cessation of exercise to assess their level of involvement in the autonomic control of cardiac rhythm in humans. Post‐exercise muscle ischemia following isometric handgrip has been shown to increase both absolute low‐frequency (aLF) power and absolute high‐frequency (aHF) power HRV (Watanabe et al., 2010) as well as the standard deviation of R‐R intervals (RRI) (Nishiyasu et al., 1994; Watanabe et al., 2010), another HRV metric, during MMA compared to baseline in healthy young adults. Further, changes in RRIs induced by MMA from post‐exercise ischemia correlate significantly with the observed changes in the standard deviation of RRIs and aHF power but were not correlated to aLF power (Watanabe et al., 2010). These findings suggest that greater PNS activity, reflected in the standard deviation of RRIs, aHF power, and to a lesser extent aLF, contributes to the control of cardiac rhythm during MMA more than SNS activity, which is mostly reflected in aLF power. However, the PNS and SNS contributions to the control of cardiac rhythm at the onset of isolated, post‐exercise MMA, that is immediately following cessation of exercise when central command and muscle mechanoreflex inputs have been removed but when the muscle metaboreflex is often still activated due to the prior exercise performance, is not well understood.

Investigations into this area have been limited by the inability to assess both SNS and PNS contributions to the control of cardiac rhythm during a short period of time and when the physiological signal is non‐stationary. Time‐domain HRV measures (root mean square of successive differences in RRIs and standard deviation of normal RRIs), reflecting PNS activity, have been shown to be higher at the onset of isolated, post‐exercise MMA following handgrip exercise in healthy male compared to female adults (Samora et al., 2020). However, SNS activity at this time remains unknown, as does PNS and SNS activity at the onset of isolated, post‐exercise MMA following lower‐limb exercise. Additionally, alterations in mechanisms regarding ANS function and cardiac control have been shown with aging, displaying a shift toward greater SNS (Esler et al., 1995) and reduced PNS (Kaye & Esler, 2008) activities. Both increased SNS and decreased PNS activity are linked with increased development of cardiovascular disease and mortality (Hasking et al., 1986; la Rovere et al., 1998; Meredith et al., 1991). Hence, it is important to investigate cardiovascular responses to physiological stimuli that acutely alter ANS activity and cardiac control in old adults to better understand how advancing age influences cardiovascular risk. Therefore, it was hypothesized that PNS activity would be restored at the onset of isolated, post‐exercise MMA following exercise in healthy young adults. Further, it was also hypothesized that PNS activity would be restored to a lesser extent at the onset of isolated, post‐exercise MMA following exercise in healthy old adults.

## 2 MATERIALS AND METHODS

### 2.1 Ethical approval

This study was based on a retrospective analysis of previously collected data. The experimental protocol in which the data were previously collected was approved by the Institutional Review Board of the Penn State Milton S. Hershey Medical Center and conformed to the Declaration of Helsinki. Following explanation of the study purpose and risks involved in the protocol, participants provided written informed consent. This protocol took place in the Clinical Research Center of Penn State Milton S. Hershey Medical Center.

### 2.2 Participants

Data from 14 young and 11 old healthy adults (Table 1) were retrospectively analyzed using CWT HRV analysis. All participants were normotensive, recreationally active, not taking any medications, and did not have any known history of cardiovascular or autonomic conditions. Participants were asked to avoid eating for 8 h, as well as ingesting caffeine or alcohol and performing exercise for 24 h prior to performing the protocol.

**TABLE 1:** Participants' demographic characteristics, baseline cardiovascular values, and maximal voluntary contraction values.

Variable	Healthy young adults	Healthy old adults
Number of participants	14	10
Age (years)	26 ± 2	62 ± 3
Sex	6 male/8 female	6 male/4 female
Racial identity/Ethnicity	12 White, NH/2 AA, NH	10 White, NH
Height (cm)	174 ± 11	174 ± 8
Weight (kg)	75 ± 16	75 ± 11
Body mass index (kg.m^−2^)	24.4 ± 2.7	24.8 ± 1.9
Systolic blood pressure (mmHg)	113 ± 9	128 ± 11
Diastolic blood pressure (mmHg)	72 ± 6	81 ± 12
Mean arterial blood pressure (mmHg)	86 ± 7	96 ± 11
Heart rate (b.min^−1^)	62 ± 8	57 ± 5
Maximum voluntary contraction (Nm)	102 ± 39	89 ± 20

*Note*: Data reported as mean ± standard deviation.

Abbreviations: AA, African American; NH, non‐Hispanic.

Baseline data for systolic BP, diastolic BP, mean arterial BP, HR, and maximal voluntary contraction (MVC) from 10 of these 14 young participants and 11 of these 11 old participants were included in analyses in previously published studies (Drew et al., 2013; Drew et al., 2015; Drew et al., 2017; Oliver et al., 2023). To date, the SNS and PNS contributions to the control of cardiac rhythm at the onset of isolated, post‐exercise MMA immediately following cessation of exercise in healthy young or old adults have yet to be investigated. The current study presents novel data on HRV parameters (aLF, aHF, nLF, nHF, and LF/HF) and RRI during baseline and all HRV parameters, HR, RRI, and mean arterial blood pressure (MAP) during the first 30 s at the onset of isolated, post‐exercise MMA immediately following cessation of exercise from all 14 young as well as 10 of the 11 old participants. Baseline data for systolic BP, diastolic BP, MAP, and HR and MVC are presented to provide context for the post‐exercise responses that were observed.

### 2.3 Experimental protocol

Participants performed two trials during a single experimental visit. Participants were seated in a semi‐supine position with their right leg flexed by 30° with the lower leg parallel to the ground and the foot strapped to a footplate to minimize heel lift during calf exercise. MVC of the right calf plantarflexor muscles was measured by recording the maximal torque that was produced when participants briefly pushed as hard as possible against the footplate. After repeating this effort three to five times, each separated by ~1 min, the largest torque recording was used as the participant's MVC. Seventy percent of each participant's MVC was calculated and set on a torque display box positioned in front of the participant, so they could visualize the level of torque produced during exercise in the 70% MVC trial.

**FIGURE 1:**
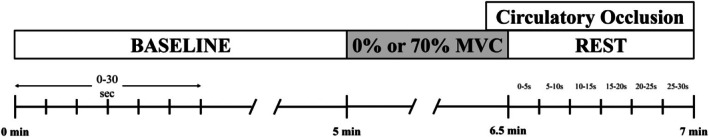
A schematic diagram of the experimental protocol showing a 5‐min resting baseline for each trial followed by either 70% maximal voluntary contraction (MVC) isometric calf exercise performance or continued rest (0% MVC) for 90 s. Data were analyzed in 5‐s time intervals during baseline and the 30 s of circulatory occlusion immediately following the rest/exercise phase of each trial, with the baseline data then averaged to produce one 30‐s average for the baseline period. MVC, maximal voluntary contraction. Timeline is not to scale.

Following identification of 70% MVC, participants were given at least 10 min after completion of the experimental set‐up. The exercise trial began with a 5‐min baseline phase (Figure 1). Following this resting phase, participants then either performed isometric right calf exercise at 70% MVC for 1.5 min (70% MVC trial) or continued to rest (0% MVC control trial). Five seconds before the end of the rest/exercise phase, a cuff around the right thigh was inflated to a supra‐systolic pressure of 250 mmHg to occlude blood flow to the lower right leg, and participants relaxed or continued resting at the end of the rest/exercise phase. This circulatory occlusion (CO) remained for 30 s following the end of the rest/exercise phase in both trials, during which time participants were relaxed. In the 70% MVC trial, this CO served to trap metabolites produced during exercise to activate the muscle metaboreflex. In the 0% MVC trial, this CO served as a control for this metabolite accumulation and consequent MMA. For all participants, the order of the two trials was counterbalanced as well as separated by ~20 min to ensure that baseline hemodynamics were restored prior to beginning the second trial.

### 2.4 Experimental measurements

Before each trial, three baseline BP measurements were taken using a semiautomated arm cuff (SureSigns VS3; Philips, Andover, MA, USA). HR was obtained from RRIs, which were continuously measured using a three‐lead electrocardiogram (Cardiocap/5; GE Healthcare, Waukesha, WI, USA). Beat‐to‐beat MAP was recorded continuously throughout all phases of both trials using finger photoplethysmography (Finometer; FMS, Arnhem, The Netherlands). Beat‐to‐beat data were calibrated offline to the mean of three baseline arm cuff measurements described previously. MAP was assessed to confirm muscle metaboreflex activation via MAP elevation above baseline levels during post‐exercise circulatory occlusion. To confirm that participants were breathing appropriately during the trials, respiratory movements were continuously measured using a pneumography belt placed around the abdomen. Torque levels produced during calf exercise were measured using a load cell on the footplate. Electrocardiogram, pneumograph, and torque signals were sampled at 1000 Hz using an analog‐to‐digital converter (Power1401; Cambridge Electronic Design (CED), Cambridge, UK; RRID:SCR_016040). Data were recorded and displayed during the trials and then analyzed offline (Spike2; CED, Cambridge, UK; RRID:SCR_000903).

### 2.5 Data and statistical analyses

CWT was applied to the RRI data for HRV analysis (MATLAB; MathWorks Inc., Natick, MA, USA; RRID:SCR_001622). Raw data files were analyzed using custom‐written script files to produce RRI and MAP values (Spike2; CED, Cambridge, UK; RRID:SCR_000903). Prior to CWT analysis, preprocessing of RRI data involved removal of ectopic beats and detrending. To obtain HRV measures using instantaneous power methods, the squared modulus of the wavelet coefficients was integrated over the desired frequency band [f1 f2]. These wavelet coefficients correspond to the specific scale factor applied to the RRI data from the ECG signal. To integrate power, frequency band wavelet scales were changed to frequencies. The values of power of each frequency band of the HRV in time were interpolated to 4 Hz, meaning frequency information was extracted from the signal instantaneously every 0.25 s (four data points per second). The instantaneous power of the frequency band [f1 f2] is given by:

$$P_{\mathrm{CWT}}\left(t\right)=\frac{1}{C_{\psi }}\int_{\alpha_{1}}^{\alpha_{2}}{\left|W\left(t,\alpha \right)\right|}^{2}\frac{\mathrm{d\alpha}}{\alpha^{2}}=\frac{1}{C_{\psi }f_{\psi }}\int_{f_{1}}^{f_{2}}{\left|W\left(t,\frac{f_{\psi }}{f}\right)\right|}^{2}\mathrm{df}$$

RRI data were analyzed using low‐frequency (LF; 0.04–0.15 Hz) and high‐frequency (HF; 0.15–0.4 Hz) bands for HRV analysis (Task Force Report, 1996). These LF and HF components are reported as log‐transformed absolute power expressed in ms^2^/Hz (aLF, aHF) and normalized values (nLF, nHF), where nLF = (aLF)/(aLF + aHF) and aHF = (aHF)/(aLF + aHF). In addition, the ratio of the aLF and aLF values was calculated (LF/HF). The first six 5‐s time windows at the start of the 5‐min baseline period were analyzed and then averaged to provide a 30‐s baseline value for analysis. This initial baseline period (0–30 s) was chosen, as opposed to the final 30 s of the 5‐min baseline period (270–300 s), to avoid the influence of anticipation of upcoming exercise performance in the 70% MVC trial causing HR to increase prior to starting exercise performance (McArdle et al., 1967). The six 5‐s time windows immediately following the end of the 1.5‐min rest/exercise phase of each trial were analyzed. As frequency information is instantaneously extracted from the signal using the CWT approach, these 5‐s time windows provide sufficient time resolution despite the brevity of their duration, highlighting the advantageous nature of this time‐frequency‐based CWT analysis approach for assessing HRV (Davrath et al., 2006).

Data are presented as mean ± standard deviation. A two‐way repeated measures analysis of variance (ANOVA) was used to determine the effect of post‐exercise MMA on cardiac autonomic control. Normality of the data for all five variables (aLF, aHF, nLF, nHF, and LF/HF) was assessed using the Shapiro–Wilk test on the studentized residuals. One participant was removed from the healthy old adult group following normality testing. Group mean and standard deviation values for data for all five variables were calculated for each time period (one baseline time period – 0–30 s, and the six, 5‐s time periods that immediately followed the end of the 1.5‐min rest/exercise phase – 0–5, 5–10, 10–15, 15–20, 20–25, and 25–30 s) in each trial (0% and 70% MVC). Posthoc analysis was conducted when main effects for trial, time, and/or an interaction between these factors were identified, which involved paired *t*‐tests with a Holm‐Bonferroni correction. Statistical significance was set at *p* < 0.05 for all analyses, and all statistical analyses were performed using SPSS Statistics for Windows (IBM, Armonk, NY, USA; RRID:SCR_016479) with figures created using Prism (GraphPad Software, San Diego, CA, USA; RRID:SCR_002798).

Additionally, while the purpose of this study was to examine the SNS and PNS contributions to the control of cardiac rhythm at the onset of isolated, post‐exercise MMA following cessation of exercise in healthy young and old adults without specifically investigating potential sex differences, data are also presented graphically disaggregated by sex to display any potential sex differences in the responses observed.

## 3 RESULTS

### 3.1 Young adults

#### 3.1.1 HR, RRI, and MAP

**FIGURE 2:**
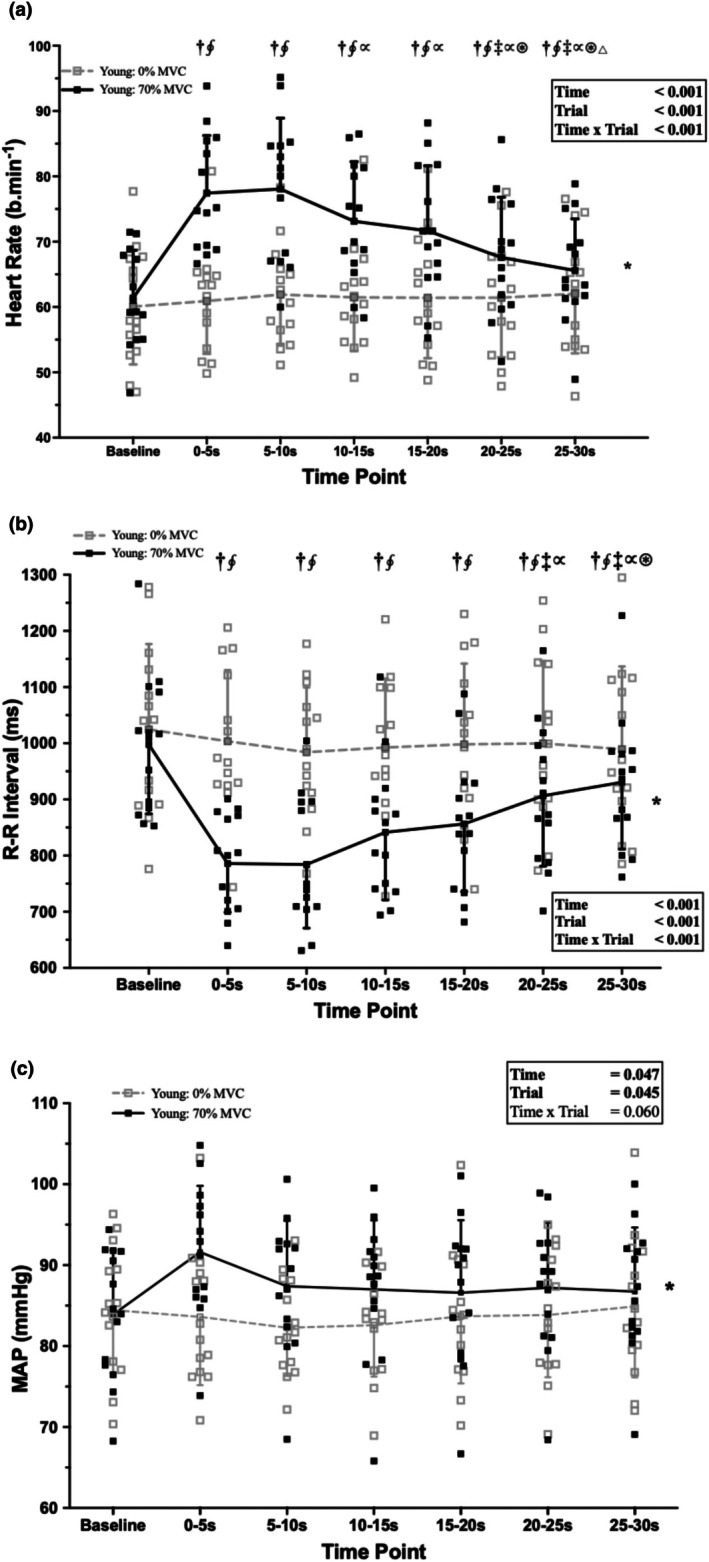
Healthy, young adults individual data and group means ± standard deviations for heart rate (HR; a), R‐R interval (b), and mean arterial pressure (MAP; c) during baseline and 30‐s of post‐exercise circulatory occlusion following one‐legged, isometric, calf exercise at 70% maximal voluntary contraction (MVC; 70% MVC trial) or continued rest (0% MVC trial). *Significantly different from 0% trial. †Significantly different from baseline (all *p* ≤ 0.018). ∮Significantly different from 0% at specific time point (all *p* ≤ 0.037). ‡Significantly different from 0 to 5 s (*p* ≤ 0.032). ∝Significantly different from 5 to 10 s (*p* ≤ 0.016). ⊛Significantly different from 10 to 15 s and 15 to 0 s (*p* ≤ 0.036). △Significantly different from 20 to 25 s (*p* = 0.024).

There were no differences in baseline values for HR, RRI, or MAP between the 0% and 70% MVC trials in the young adults (*p* = 0.326, *p* = 0. 289, and *p* = 0.786, respectively). There was a significant time × trial interaction as well as effects of time and trial for HR (Time: *p* < 0.001, Trial: *p* < 0.001, Time × Trial: *p* < 0.001; Figure 2a) and RRI (Time: *p* < 0.001, Trial: *p* < 0.001, Time × Trial: *p* < 0.001; Figure 2b) in the young adults. HR was significantly higher during the six MMA timepoints (0–30 s) compared to baseline and the corresponding timepoints in the 0% trial (*p* ≤ 0.017 for all). HR was highest during the first two MMA timepoints (0–10 s; *p* ≤ 0.012 for both) and then progressively decreased toward the baseline level by the last MMA timepoint (25–30 s; *p* = 0.014). RRI was significantly lower during the six MMA timepoints (0–30 s) compared to baseline and the corresponding timepoints in the 0% trial (*p* ≤ 0.037 for all). RRI was lowest during the first two MMA timepoints (0–10 s; *p* ≤ 0.014) and then progressively increased toward the baseline level by the last MMA timepoint (25–30 s; *p* = 0.013). There were significant main effects of time and trial for MAP (Time: *p* = 0.047, Trial: *p* = 0.045, Time × Trial: *p* = 0.060; Figure 2c) in the young adults. MAP was higher during the 70% trial compared to the 0% trial.

#### 3.1.2 HRV

**FIGURE 3:**
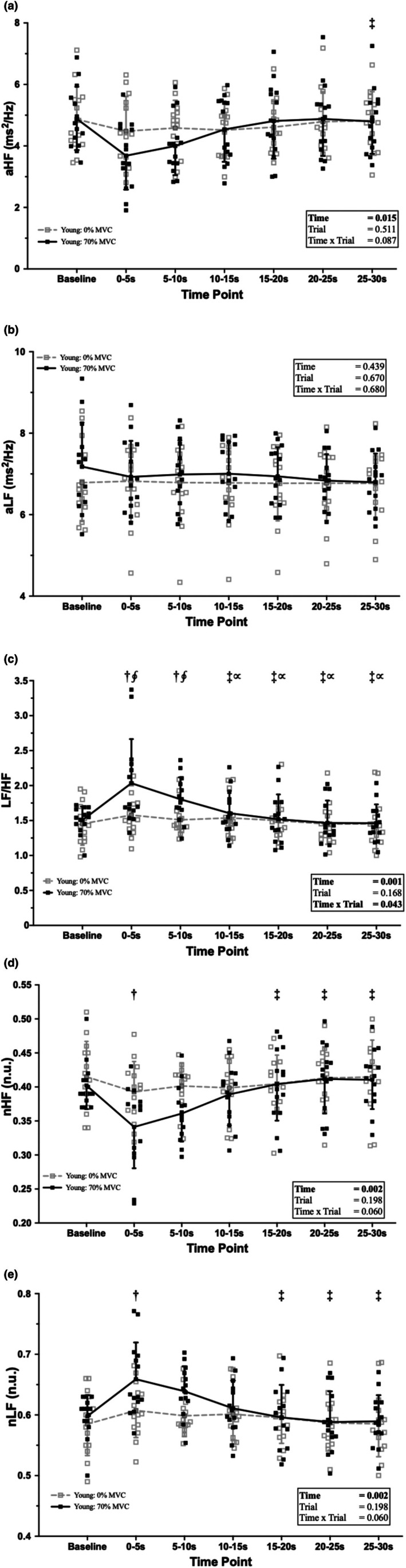
Healthy, young adults individual data and group means ± standard deviations for absolute high frequency (aHF; a) power, absolute low‐frequency power (aLF; b), the LF/HF ratio (c), normalized high‐frequency (nHF; d) and normalized low‐frequency (nLF; e) during baseline and 30‐s of post‐exercise circulatory occlusion following one‐legged, isometric, calf exercise at 70% maximal voluntary contraction (MVC; 70% MVC trial) or continued rest (0% MVC trial). N.u., normalized units. †Significantly different from baseline (*p* < 0.05). ∮Significantly different from 0% at specific time point (all *p* < 0.05). ‡ Significantly different from 0 to 5 s (*p* < 0.05). ∝Significantly different from 5 to 10 s (*p* < 0.05).

There were no differences in baseline values for aHF, aLF, LF/HF, nHF, and nLF between the 0% and 70% MVC trials in the young adults (*p* = 0.929, *p* = 0.187, *p* = 0.458, *p* = 0.291, and *p* = 0.291, respectively). There was a significant effect of time for aHF in the young adults (Time: *p* = 0.015, Trial: *p* = 0.511, Time × Trial: *p* = 0.087; Figure 3a). The last MMA timepoint (25–30 s) was significantly different from the first MMA timepoint (0–5 s; *p* = 0.042), which appears to be due to the lower aHF during the first MMA timepoint (0–5 s) in the 70% trial. There were no significant differences for aLF in the young adults (Time: *p* = 0.670, Trial: *p* = 0.439, Time × Trial: *p* = 0.680; Figure 3b). There was a significant time × trial interaction as well as an effect of time for LF/HF in the young adults (all *p* < 0.001; Figure 3c). LF/HF was significantly higher during the first two MMA timepoints (0–10 s) compared to baseline and the corresponding timepoints in the 0% trial (*p* ≤ 0.036 for all). LF/HF then decreased back to the baseline level in the last four MMA timepoints (10–30 s). There was a significant effect of time for nHF in the young adults (Time: *p* = 0.002, Trial: *p* = 0.198, Time × Trial: *p* = 0.06; Figure 3d). nHF was significantly lower during the first MMA timepoint (0–5 s) compared to baseline and then progressively increased back to the baseline level by the last three MMA timepoints (15–30 s). There was a significant effect of time for nLF in the young adults (Time: *p* = 0.002, Trial: *p* = 0.198, Time × Trial: *p* = 0.06; Figure 3e). nLF was significantly higher during the first MMA timepoint (0–5 s) compared to baseline and then progressively decreased back to the baseline level by the last three MMA timepoints (15–30 s).

### 3.2 Old adults

#### 3.2.1 HR, RRI, and MAP

**FIGURE 4:**
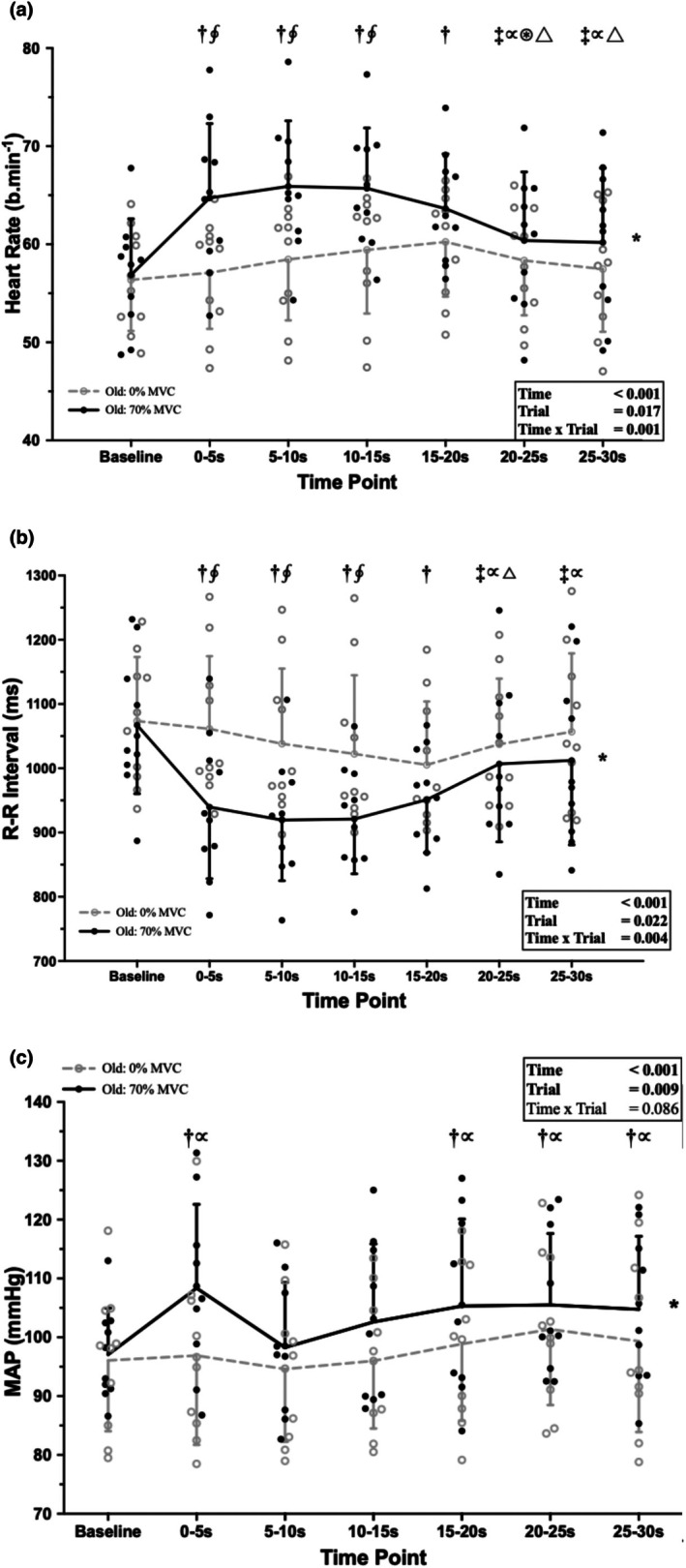
Healthy, old adults individual data and group means ± standard deviations for heart rate (HR; a), R‐R intervals (b), and mean arterial pressure (MAP; c) during baseline and 30‐s of post‐exercise circulatory occlusion following one‐legged, isometric, calf exercise at 70% maximal voluntary contraction (MVC; 70% MVC trial) or continued rest (0% MVC trial). *Significantly different from 0% trial. †Significantly different from baseline (all *p* ≤ 0.021). ∮Significantly different from 0% at specific time point (all *p* ≤ 0.018). ‡ Significantly different from 0 to 5 s (*p* ≤ 0.027). ∝Significantly different from 5 to 10 s (*p* ≤ 0.035). ⊛ Significantly different from 10 to 15 s (*p* ≤ 0.035). △Significantly different from 15 to 20 s (*p* ≤ 0.042).

There were no differences in baseline values for HR, RRI, or MAP between the 0% and 70% MVC trials in the old adults (*p* = 0.61, *p* = 0.72, and *p* = 0.604, respectively). There was a significant time × trial interaction as well as effects of time and trial for HR (Time: *p* < 0.001, Trial: *p* = 0.017, Time × Trial: *p* = 0.001; Figure 4a) and RRI (Time: *p* < 0.001, Trial: *p* = 0.022, Time × Trial: *p* = 0.004; Figure 4b) in the old adults. HR was significantly higher during the first four MMA timepoints (0–20 s) compared to baseline (*p* ≤ 0.02 for all) and the first three MMA timepoints (0–15 s) compared to the corresponding timepoints in the 0% trial (*p* ≤ 0.014 for all). HR decreased toward baseline levels by the last two MMA timepoints (20–30 s; *p* ≤ 0.036 for all). RRI was significantly lower during the first four MMA timepoints (0–20 s) compared to baseline (*p* ≤ 0.03 for all) and the first three MMA timepoints (0–15 s) compared to the corresponding timepoints in the 0% trial (*p* ≤ 0.018 for all). RRI then increased toward baseline levels by the last two MMA timepoints (20–30 s; *p* ≤ 0.042 for all). There were significant effects of time and trial for MAP (Time: *p* < 0.001, Trial: *p* = 0.009, Time × Trial: *p* = 0.086; Figure 4c) in the old adults. MAP was higher during the first and final three MMA timepoints (0–5 s, 15–30 s) compared to baseline (*p* ≤ 0.021 for all). Overall, MAP was higher in the 70% MVC trial compared to the 0% MVC trial.

#### 3.2.2 HRV

**FIGURE 5:**
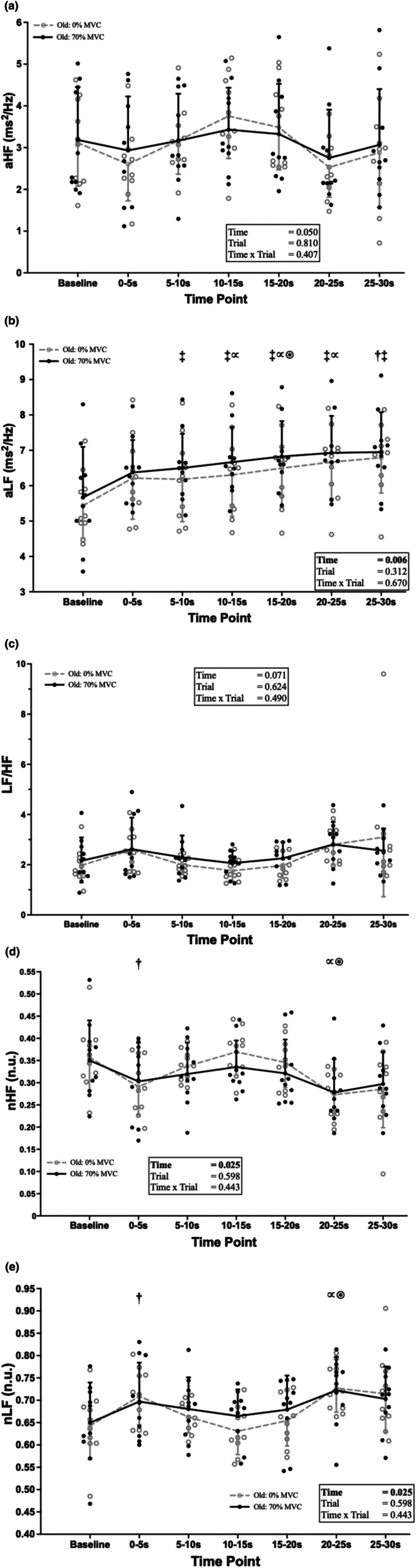
Healthy, old adults individual data and group means ± standard deviations for absolute high frequency (aHF; a) power, absolute low‐frequency power (aLF; b), the LF/HF ratio (c), normalized high‐frequency (nHF; d), and normalized low‐frequency (nLF; e) during baseline and 30‐s of post‐exercise circulatory occlusion following one‐legged, isometric, calf exercise at 70% maximal voluntary contraction (MVC; 70% MVC trial) or continued rest (0% MVC trial). N.u., normalized units. †Significantly different from baseline (all *p* < 0.05). ‡ Significantly different from 0 to 5 s (all *p* < 0.05). ∝Significantly different from 5 to 10 s (all *p* < 0.05). ⊛ Significantly different from 10 to 15 s (all *p* < 0.05).

There were no differences in baseline values for aHF, aLF, LF/HF, nHF, and nLF between the 0% and 70% MVC trials in the old adults (*p* = 0.681, *p* = 0.414, *p* = 0.427, *p* = 0.702, and *p* = 0.702, respectively). There were no significant differences for aHF in the old adults (Time: *p* = 0.05, Trial: *p* = 0.81, Time × Trial: *p* = 0.407; Figure 5a). There was a significant effect of time for aLF in the old adults (Time: *p* = 0.006, Trial: *p* = 0.312, Time × Trial: *p* = 0.67; Figure 5b). The last MMA timepoint (25–30 s) was higher compared to baseline (*p* = 0.034), and the last five MMA timepoints (5–30 s) were higher than the first MMA timepoint (0–5 s; *p* ≤ 0.049 for all). There were no significant differences for LF/HF in the old adults (Time: *p* = 0.071, Trial: *p* = 0.624, Time × Trial: *p* = 0.49; Figure 5c). There was a significant effect of time for nHF in the old adults (Time: *p* = 0.025, Trial: *p* = 0.598, Time × Trial: *p* = 0.443; Figure 5d). nHF was lower during the first MMA timepoint (0–5 s) compared to baseline (*p* = 0.014), and the fifth MMA timepoint (20–25 s) was lower than the second, third, and fourth MMA timepoints (5–20 s; *p* ≤ 0.036 for all). There was a significant effect of time for nLF in the old adults (Time: *p* = 0.025, Trial: *p* = 0.598, Time × Trial: *p* = 0.443; Figure 5e). nLF was higher during the first MMA timepoint (0–5 s) compared to baseline (*p* = 0.014), and the fifth MMA timepoint (20–25 s) was higher than the second, third, and fourth MMA timepoints (5–20 s; *p* ≤ 0.036 for all).

### 3.3 Data disaggregated by sex

**FIGURE 6:**
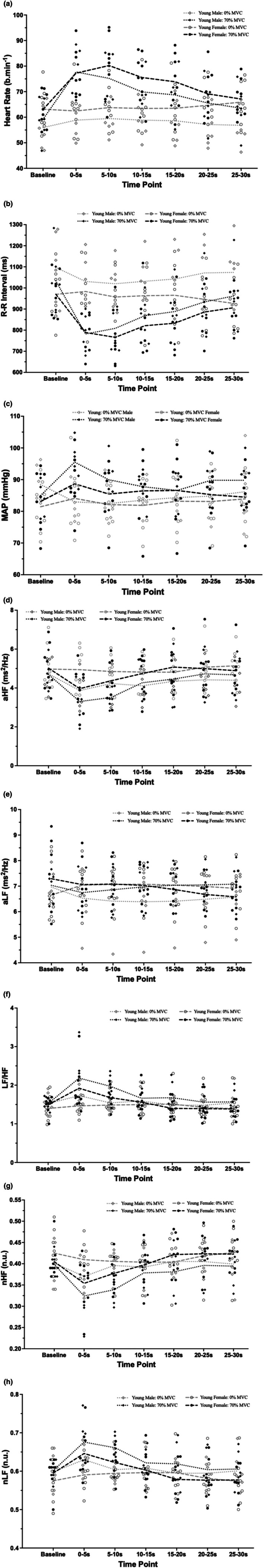
Healthy, young adults' individual data and group means ± standard deviations for heart rate (HR; a), R‐R intervals (b), mean arterial pressure (MAP; c), absolute high‐frequency (aHF; d) power, absolute low‐frequency (aLF; e) power, the LF/HF ratio (f), normalized high‐frequency (nHF; g), and normalized low‐frequency (nLF; h) during baseline and 30‐s of post‐exercise circulatory occlusion following one‐legged, isometric calf exercise at 70% maximal voluntary contraction (MVC; 70% MVC trial) or continued rest (0% MVC trial) disaggregated by sex. N.u., normalized units.

**FIGURE 7:**
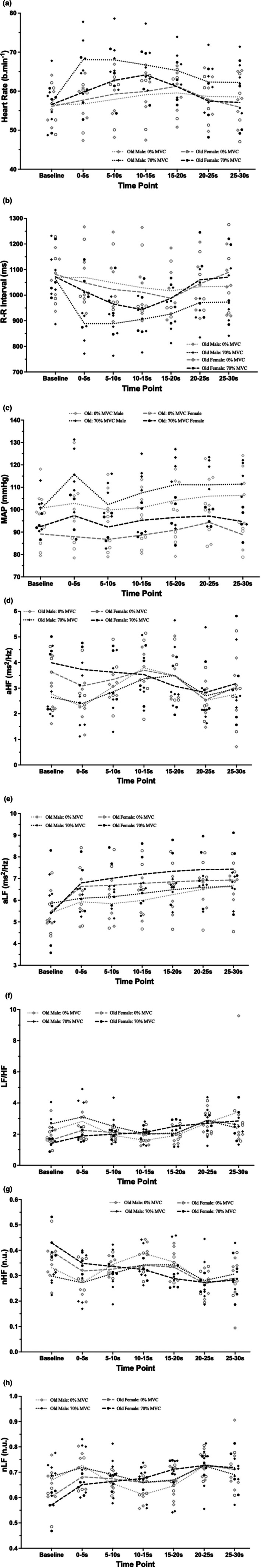
Healthy, old adults' individual data and group means ± standard deviations for heart rate (HR; a), R‐R intervals (b), mean arterial pressure (MAP; c), absolute high‐frequency (aHF; d) power, absolute low‐frequency (aLF; e) power, the LF/HF ratio (f), normalized high‐frequency (nHF; g), and normalized low‐frequency (nLF; h) during baseline and 30‐s of post‐exercise circulatory occlusion following one‐legged isometric calf exercise at 70% maximal voluntary contraction (MVC; 70% MVC trial) or continued rest (0% MVC trial) disaggregated by sex. N.u., normalized units.

HR, RRI, MAP, aHF, aLF, LF/HF, nHF, and nLF data disaggregated by sex in the young adults are presented in Figure 6. HR, RRI, MAP, aHF, aLF, LF/HF, nHF, and nLF data disaggregated by sex in the old adults are presented in Figure 7.

## 4 DISCUSSION

The present study aimed to investigate the autonomic control of cardiac rhythm at the onset of MMA following isometric calf exercise assessed by time‐frequency‐based HRV analysis in healthy young and old adults. Findings from this study are that healthy young adults exhibit restoration of parasympathetic control of cardiac rhythm at the onset of MMA following 70% MVC isometric exercise (Figure 3a,c,d). In contrast, this restoration of parasympathetic control of cardiac rhythm at the onset of MMA following 70% MVC isometric exercise is absent in healthy old adults (Figure 5c).

In young adults, within the first 5 s of MMA following 90‐s, 70% MVC one‐legged, isometric, calf exercise, HR, LF/HF, and nLF were increased and RRI, aHF, and nHF were decreased (Figures 2 and 3). These changes would be due to muscle mechanoreflex activation and central command during the exercise that was performed inhibiting cardiac vagal outflow from the nucleus tractus solitarii (NTS) in the medulla oblongata, resulting in parasympathetic withdrawal (Drew, 2017; Fisher et al., 2015). Parasympathetic withdrawal decreased RRI, therefore increasing HR, and decreased aHF, which would then explain the higher LF/HF, lower nHF, and higher nLF within the first 5 s of MMA following exercise performance.

Over the 30‐s MMA, RRI increased (Figure 2b) and HR decreased (Figure 2a) progressively toward baseline levels but remained slightly above baseline levels by the last 5 s of MMA in young adults. This is in line with findings from previous investigations in which HR remained elevated during circulatory occlusion following isometric handgrip exercise (Fisher et al., 2010), dynamic leg cycling (Fisher et al., 2013), and high‐intensity isometric leg exercise (Drew, Bell, & White, 2008; Drew, McIntyre, et al., 2008). MAP was higher during the 70% trial compared to the 0% trial in the young adults, indicating post‐exercise muscle metaboreflex activation. Over the 30‐s MMA, parasympathetic activity, as indicated by aHF (Figure 3a) and nHF (Figure 3d), progressively returned to baseline levels, as did LF/HF (Figure 3c) and nLF (Figure 3e). There were no significant changes in aLF during MMA (Figure 3b), indicating no significant changes in sympathetic activity, which emphasizes the predominance of changes in PNS activity that affected cardiac control during MMA. This restoration of parasympathetic activity during the onset of MMA would be due to the cessation of muscle mechanoreflex activation and central command at the end of exercise and the consequent removal of these inhibitory inputs to the NTS that would allow greater cardiac vagal outflow. These findings are in alignment with some previous work regarding PNS activity returning to the baseline level during MMA (Iellamo et al., 1999) but contrast with some prior findings showing augmented PNS activity during MMA (Fisher et al., 2010) and SNS activity remaining elevated above the baseline level during MMA (Iellamo et al., 1999). The differences in these findings could be due to different durations of MMA, different intensities, muscle groups involved, and/or durations of exercise preceding MMA, the use of time‐frequency‐, frequency‐, or time‐based HRV variables, and/or whether male and female participants were studied or only male participants. In addition, with exercise performance increasing BP, MMA following exercise partially maintains BP at a level elevated above baseline, causing a baroreflex‐mediated increase in cardiac vagal outflow during MMA. These combined neural adjustments drive the rapid changes in autonomic control of cardiac rhythm during the onset of MMA, with PNS control playing a predominant role.

In old adults, within the first 5 s of MMA following 90‐s, 70% MVC one‐legged isometric calf exercise, HR and nLF were increased and RRI and nHF were decreased, but aHF, aLF, and LF/HF were not different to baseline (Figures 4 and 5). These findings are in contrast with what was observed in young adults. The parasympathetic withdrawal occurring during the exercise that was performed due to the inhibitory inputs caused by muscle mechanoreflex activation and central command took place to a lesser extent in the old adults compared to the young adults. This is demonstrated by the HR increase of ~16 b.min^−1^ in young adults (Figure 2a) but only ~8 b.min^−1^ in old adults (Figure 4a). This age‐related difference in HR response has similarly been shown during isometric handgrip exercise (Clark et al., 2016; Houssiere et al., 2006; Muller et al., 2012; Ng et al., 1994; Taylor et al., 1991) and is due to lower overall PNS activity (Stratton et al., 2003) and/or reduced cardiac M_2_ muscarinic receptor density and function (Brodde et al., 1998; Poller et al., 1997) with advancing age.

Over the 30‐s MMA, RRI increased (Figure 4b) and HR decreased (Figure 4a) progressively toward baseline levels and returned to baseline levels by 20–30 s of MMA in old adults. The faster return of RRI and HR to baseline levels in old adults compared to young adults is likely due to the smaller changes in RRI and HR during the previously performed exercise in old adults compared to young adults. MAP was elevated above the baseline level during MMA timepoints in the 70% trial and was higher during the 70% trial compared to the 0% trial in the old adults, indicating post‐exercise muscle metaboreflex activation. The unchanged aHF within the first 5 s of MMA following exercise in old adults (Figure 5a) further suggests that old adults experienced less parasympathetic withdrawal during exercise than young adults. This finding during MMA is in agreement with recent work in which older adults displayed less parasympathetic withdrawal, as assessed using time‐domain HRV, than young adults during MMA immediately following isometric handgrip exercise (Sabino‐Carvalho et al., 2025). This unchanged aHF within the first 5 s of MMA following exercise in old adults also explains the unchanged LF/HF at this time (Figure 5c), as aLF was also unchanged within the first 5 s of MMA following exercise (Figure 5b). Consequently, aHF and LF/HF remain unchanged over the 30‐s MMA, demonstrating that old adults do not exhibit parasympathetic restoration during the onset of MMA following exercise, which contrasts with what occurs in young adults.

nHF was lower (Figure 5d) and nLF was higher (Figure 5e) within the first 5 s of MMA following exercise in old adults, but these changes occurred in both the 70% and 0% MVC trials, suggesting that these were not responses related to exercise performance. There was a small, gradual increase in aLF over the 30‐s period following exercise (Figure 5b), but this also occurred in both the 70% and 0% MVC trials, also suggesting that these were not responses related to exercise performance. It is unclear why there was a small, gradual increase in aLF in the old adults. It is possible that this was an effect of cuff inflation, as a similar response was seen in both the 0% and 70% trials in the old adults. While cuff inflation can induce pain in young adults (Williamson et al., 1994), its effect in old adults is unknown. Alternatively, the old adults exhibited relatively small changes from baseline in all HRV variables. In the absence of larger changes, such as the larger changes seen in the young adults, this could potentially lead to a statistical difference being observed even though the physiological significance of this difference may be minimal.

There are some limitations to consider for this study. While respiratory movement was measured, respiratory rate and tidal volume were not controlled, as participants breathed spontaneously throughout the trials. Previous studies have shown that ventilation returns to baseline levels during circulatory occlusion following 40%–50% MVC isometric handgrip exercise (Bruce & White, 2012; Lykidis et al., 2010). This suggests that ventilation would have had minimal influence on the responses observed in the 70% MVC compared to the 0% trials in this study, and spontaneous breathing represents a typical breathing pattern of individuals. Whether pain occurred during circulatory occlusion via thigh‐cuff inflation was not assessed. While thigh‐cuff inflation can induce pain (Williamson et al., 1994), the thigh‐cuff inflation applied in the current study was to a lesser extent than when pain occurred in an earlier study (Williamson et al., 1994). We applied a lower pressure (250 vs. 300 mmHg), only one cuff instead of two, and for a shorter duration (30 s vs. 2 or more minutes). Therefore, it is less likely that pain was experienced during cuff inflation in the current study. The influence of external mechanical compression through cuff inflation on cardiovascular control is a consideration. As previously mentioned, the small, gradual increase in aLF in the 0% trial in the old adults was an unexpected observation. A potential explanation for this observation is activation of the muscle mechanoreflex from external compression of the thigh cuff. While it has been shown that external mechanical pressure applied to skeletal muscle can increase blood pressure (Williamson et al., 1994), its influence on heart rate appears minimal (Williamson et al., 1994). In the current study, heart rate and blood pressure did not change from baseline levels during the circulatory occlusion phase in the 0% trial. Therefore, while an effect of the occlusion alone on blood pressure or heart rate in our study cannot be discounted, this influence appears minimal. Body position may have affected sympathetically mediated cardiac responses to post‐exercise circulatory occlusion due to changes in central blood volume distribution and total peripheral resistance (Teixeira et al., 2018). Participants in this study were seated in a semi‐supine position, which, relative to a supine position, decreases central blood volume and load on cardiopulmonary baroreceptors and results in increased sympathetic outflow (Teixeira et al., 2018). However, participants' semi‐supine body position would be more representative of a seated position that individuals adopt more on a daily basis than a supine position. HRV responses during the exercise preceding isolated muscle metaboreflex activation were not shown. While HRV responses during and following exercise, with and without muscle metaboreflex activation, warrant study, the focus of the current study was specifically on HRV responses during the onset of muscle metaboreflex activation following exercise in healthy young adults. This work expands our previous work examining the autonomic control of cardiac rhythm specifically at the onset of exercise in healthy young adults (Oliver et al., 2023). Therefore, the novelty of our current findings is the characterization of the autonomic control of cardiac rhythm at the onset of muscle metaboreflex activation following exercise in populations of high parasympathetic activity (healthy young adults) and low parasympathetic activity (healthy old adults).

## 5 CONCLUSIONS

The findings from this study suggest that healthy young adults exhibit restoration of PNS control of cardiac rhythm at the onset of MMA following exercise. These results emphasize the predominance of changes in PNS activity that affect cardiac control during the onset of MMA. Conversely, this restoration of PNS control of cardiac rhythm at the onset of MMA following exercise is absent in healthy old adults. In addition to the known reductions in PNS activity with advancing age, aging appears to diminish cardiac vagal reactivity following exercise. Future work is needed to more closely examine the mechanisms involved in these autonomic changes, particularly with regard to aging.

## AUTHOR CONTRIBUTIONS

**Tyler E. Oliver:** Data curation; formal analysis; investigation; validation; visualization. **Miguel E. Sánchez‐Hechavarría:** Investigation; resources. **Ramón Carrazana‐Escalona:** Investigation; resources. **Cheryl A. Blaha:** Resources. **Lawrence I. Sinoway:** Funding acquisition. **Rachel C. Drew:** Conceptualization; data curation; formal analysis; investigation; methodology; project administration; resources; supervision; validation; visualization.

## FUNDING INFORMATION

This study was supported by National Institutes of Health grants P01 HL096570 (LIS) and UL TR000127 (LIS).

## CONFLICT OF INTEREST STATEMENT

The authors declare no competing interests.

## Data Availability

The de‐identified, numerical datasets used and/or analyzed for this study are available from the corresponding author upon reasonable request.
